# Small cell neuroendocrine carcinoma of the cervix: insights from a state cancer institute in India

**DOI:** 10.3332/ecancer.2026.2109

**Published:** 2026-04-07

**Authors:** Nishu Dhingra, Dama Swetha, Pariseema S Dave, Bijal M Patel, Chetna Parekh, Ruchi Arora

**Affiliations:** 1 Department of Obstetrics and Gynaecology, AIIMS, Bhatinda 151001, India; 2 Department of Gynaecological Oncology, The Gujarat Cancer and Research Institute, Ahmedabad, Gujarat 380016, India

**Keywords:** neuroendocrine tumours, chromogranin A, synaptophysin, neuron-specific enolase, etoposide, cisplatin, prophylactic cranial irradiation

## Abstract

**Background:**

Small-cell neuroendocrine carcinoma of the cervix (NECC) is a rare and aggressive malignancy, accounting for 0.5%–1% of invasive cervical cancers. It is marked by rapid progression, early nodal and distant spread, and poor survival even in early stages. Management is challenging in low- and middle-income countries (LMIC) due to delayed diagnosis and advanced presentation. This study describes the clinicopathological features, treatment approaches and outcomes from a state cancer institute in India.

**Methods:**

We retrospectively analysed data of women with biopsy-proven NECC treated between January 2004 and December 2020 at a tertiary state cancer centre. Clinical, pathological and treatment data were extracted. Immunohistochemistry confirmed all cases. Survival was assessed using Kaplan–Meier analysis.

**Results:**

Of 54 patients identified, 32 were included for analysis. Median age was 51.2 years (range 30–77). Most patients were multiparous, 87.5% illiterate and 31.2% tobacco users. Advanced disease presentation noted, 68.7% with stage III and 12.5% with stage IV. Tumour size >4 cm was noted in 81.2%, pelvic nodes in 53.1% and para-aortic nodes in 29%. Treatment included concurrent chemoradiation (n = 19), surgery and adjuvant chemoradiation (n = 5), neoadjuvant chemotherapy followed by chemoradiation (n = 5) and palliative therapy (n = 3). Platinum–etoposide was the most frequently used chemotherapy regimen. Median time to recurrence was 9 months; pelvic and distant metastases were common. Four-year overall survival was 24.7%. Only two long-term survivors were observed, both stage III patients treated with intensive multimodality therapy.

**Conclusion:**

NECC demonstrates aggressive clinical behaviour with poor survival despite multimodality treatment. Early diagnosis, timely systemic therapy and collaborative multicentre data are essential to improve outcomes, especially in LMIC settings.

## Introduction

Neuroendocrine carcinoma of the cervix (NECC) is a rare and aggressive form of cervical cancer, accounting for 0.5%–1% of all invasive cervical malignancies [1]. These tumours tend to grow rapidly, metastasise early and often present at an advanced stage, making timely diagnosis and treatment a significant challenge, especially in low-resource settings.

The World Health Organisation's 2020 classification groups neuroendocrine tumours (NETs) of the cervix into two broad categories: neuroendocrine tumours, which include carcinoid and atypical carcinoid tumours and neuroendocrine carcinomas (NECs), which include small-cell and large-cell variants [2]. While NETs are extremely rare in the cervix, small-cell NEC remains the most frequently encountered and clinically aggressive subtype.

Most cases of NECC are associated with high-risk human papillomavirus (HPV), particularly HPV-18 [3–6]. The origin is thought to be from multi-potent reserve cells of the endocervical epithelium, which may undergo neuroendocrine transformation under certain stimuli [7]. These tumours are characterised by distinct histological features and are supported by immunohistochemical markers such as chromogranin A, synaptophysin and CD56 [8].

Although treatment guidelines are available, they are largely based on small retrospective series and expert consensus, with prospective evidence remaining scarce. In real-world practice, particularly in low- and middle-income countries (LMIC) such as India, delayed diagnosis, limited access to advanced imaging, immunohistochemistry (IHC) and presentation with bulky or metastatic disease add further challenges to management.

At our institution, we retrospectively reviewed all small cell NECC cases treated over 16 years to understand clinical behaviour, management patterns and survival outcomes in a real-world, resource-constrained setting.

## Material and methods

This is a retrospective, single-institution study conducted at The Gujarat Cancer & Research Institute, Ahmedabad, after approval from the Institutional Review Board.

We included patients diagnosed with small cell NECC between January 2004 and December 2020 and were followed up till June 2024. We collected data on patient demographics (age, parity, literacy and smoking history), clinical details (presenting symptoms, tumour size, International Federation of Obstetrics and Gynecology (FIGO) 2018 stage and nodal involvement), treatment modalities (surgery, chemotherapy, radiotherapy (RT), including prophylactic cranial irradiation (PCI) and follow-up).

Histological diagnosis was confirmed with IHC. Panel of IHC markers: synaptophysin, chromogranin A, neuron-specific enolase (NSE), cytokeratin, CD56, EMA, S-100 and Ki-67 were used.

Follow-up was scheduled every 3 months for the first 2 years, and every 6 months thereafter, with symptom evaluation, pelvic exam and imaging (ultrasound). In the event of any clinical or radiological suspicion, additional imaging tests were done.

Survival analysis was performed using the Kaplan–Meier method. Progression-free survival and overall survival (OS) were compared using the log-rank test. Descriptive statistics were applied using SPSS version 22.

## Results

Out of 54 patients diagnosed with small-cell NECC at our centre between 2004 and 2020, 32 fulfilled the inclusion criteria for analysis. The remaining 22 were excluded, 13 because of incomplete records and nine were lost to follow-up.

The median age at diagnosis was 51.2 years, ranging from 30 to 77 years. Most women were multiparous, 87.5% were illiterate and around one-third (31.2%) had a history of tobacco use.

The most common symptom was vaginal discharge (90%), followed by abdominal pain (53.3%) and postmenopausal bleeding (50%). Post-coital bleeding reported by two patients and one patient presented with deep vein thrombosis. Most patients were staged using contrast-enhanced CT scans, as the availability of MRI or PET-CT was limited. Patient characteristics are tabulated in Table 1.

### Tumour characteristics

Most patients presented with advanced-stage disease. Four patients were diagnosed at an early stage (Stage I or II), while the majority (68.7%) were Stage III and 12.5% were Stage IV. Two patients had vaginal vault recurrence after surgery performed elsewhere and adjuvant treatment was not taken. Tumour size was more than 4 cm in 26 out of 32 cases (81.2%). Pelvic lymph node involvement was seen in 53.1%, and para-aortic nodes in 29% of cases.

On IHC, synaptophysin and chromogranin A were the most commonly positive markers, consistent with neuroendocrine features. A few tumours also showed positivity for NSE, cytokeratin and CD56. Tumour characteristic described in described in Table 2.

### Treatment modalities and response

Treatment varied depending on the stage at diagnosis and patient performance status, where initial care was received.

## Primary surgery with or without adjuvant therapy (n = 5)

Two patients underwent surgery followed by adjuvant therapy at our centre. One had stage IB3 disease, received adjuvant chemoradiation and PCI, with an OS of 12 months. The other patient, with stage IB2, underwent radical hysterectomy and adjuvant therapy, remained disease-free for 45 months and died without documented recurrence.

The remaining three patients had surgery performed at outside centres. Two of them (Stage I and Stage IIIC2r) did not receive adjuvant therapy and later presented to our centre with vault recurrence. The fifth patient (Stage IIIC1p) presented to us for adjuvant therapy after surgery and was lost to follow-up after completing treatment. Treatment details and outcome are described in Table 3.

## Neoadjuvant chemotherapy followed by chemo-radiation (n = 5)

Five patients received neoadjuvant chemotherapy (NACT) as the initial treatment. These patients had locally advanced disease, ineligible for upfront curative chemoradiation or had high disease burden, including metastases. All belonged to Stage III or IV. Two patients received RT after chemotherapy, both of whom also received PCI. One patient received palliative (haemostatic) pelvic RT due to bleeding from the cervical lesion and PCI. Two patients received only NACT and one of them died within 2 months and the other was lost to follow up. Patient treatment details and outcomes are described in Table 4.

## Concurrent chemoradiation (n = 219)

This was the most used treatment approach, as most patients presented with Stage III disease. All patients in this group received pelvic external beam RT (EBRT) with weekly concurrent cisplatin and etoposide, followed by brachytherapy. Stage-wise distribution in this group is tabulated in Table 5.

Out of 19 patients who received definitive concurrent chemoradiation (CCRT), 11 (57.8%) developed recurrence. The pelvis was the most common site of failure. Some patients also developed distant metastases: one patient with Stage IIIC2r developed brain and vertebral metastases. She received whole brain RT and RT to the spine. Later, she developed pelvic recurrence with rectal infiltration and was treated with four cycles of etoposide and cisplatin. Another patient with Stage IIIB developed metastases to the scapula, vertebrae and liver, and received palliative RT to the involved sites. One patient with Stage IIIB had adrenal metastasis and received six cycles of paclitaxel and etoposide.

At the last follow-up, only two patients in this group were alive. Both belonged to Stage III: one received 50.4 Gy of pelvic EBRT with concurrent cisplatin and etoposide, brachytherapy and PCI (26 Gy in 13 fractions). Her OS was 16 years. The second patient, with Stage III disease, completed CCRT and additional chemotherapy and was alive with an OS of 80 months.

## Palliative therapy (n = 3)

Three patients received treatment with palliative intent. One patient with Stage IIIB disease presented with femoral vein thrombosis and brain metastases. Another patient had Stage IVB disease with fine needle aspiration cytology-confirmed supraclavicular lymph node involvement. The third patient had Stage IIIC1r disease with poor performance status (Eastern Cooperative Oncology Group (ECOG)) and was given palliative RT for local symptom control. Treatment details are mentioned in Table 6.

### Survival analysis

Factors showing statistical significance after univariate analysis is shown in Table 7 and after multivariate analysis is shown in Table 8. Only Parity ≥3 met the *p* < 0.2 threshold for multivariate inclusion. Age was also included due to clinical relevance.

The median follow-up duration was 42.6 months (range: 1.5–238.2 months), calculated using reverse Kaplan-Meier methodology, with censoring applied at the last follow-up date (31 December 2024) for patients who were alive. Out of 32 patients, only two were alive at the last follow-up. The median follow-up was 42.6 months (range, 1.5–238.2 months). Four-year OS is 24.7%.

### KM curves for OS

Figure 1a–c, KM curves for survival by age, tumour size, FIGO stage.

## Discussion

The median age at diagnosis was 51.2 (30–77) years, most patients were multiparous and over two-thirds (68.7%) presented with stage III disease. Tumour size exceeded 4 cm in 81.2%, pelvic nodal involvement was present in 53.1% and para-aortic nodal metastases in 29%. Treatment was heterogeneous, based on stage at diagnosis, performance status and resource constraints. Etoposide–cisplatin-based chemotherapy, delivered either as CCRT or as part of multimodality therapy, was the most used systemic regimen. PCI was administered to five patients at the clinician's discretion. OS was poor, with a 4-year OS of 24.7%. None of the patients with stage I disease survived at the last follow-up. Only two patients, both with stage III disease and treated with intensive multimodality therapy – achieved long-term survival (80 months and 16 years, respectively). Median time to recurrence was 9 months, with pelvic and distant metastases being the most common patterns of failure.

### Comparison with existing literature

Globally, NECC is recognised as a rare and aggressive cervical malignancy [1, 2]. Our stage distribution correlated with other Indian series [9] and international reports [10, 11]. Early-stage NECC has been associated with better survival in some studies, with reported 5-year OS ranging from 30% to 63% [10, 11]. However, our finding of no survivors among stage I patients is comparable to Abeler *et al* [12], who reported a 5-year survival of only 14% [13, 14]. Chemotherapy, particularly platinum–etoposide, is considered essential in NECC due to its propensity for early hematogenous spread [15–18]. In our study, E-P was the primary chemotherapy regimen, consistent with Hoskins *et al* [19], who reported high failure-free survival in early-stage patients treated with EP-based CCRT. In our study, the two long-term survivors were both stage III patients who received intensive multimodality therapy, suggesting that in selected advanced-stage patients, aggressive combined treatment may alter the disease course. Some centres have explored NACT followed by CCRT for bulky or advanced disease, with encouraging results. Our immunohistochemical profile, synaptophysin (51.8%), chromogranin A (48.1%) and variable expression of NSE and cytokeratin is comparable to prior reports [9].

The poor outcomes in our series reflect both the aggressive biology of NECC and the realities of care in LMIC. Limited access to advanced imaging and IHC delays accurate staging and diagnosis. Many patients present late, often after initial evaluation in peripheral centres. Treatment protocols are adapted from small cell lung cancer and systemic therapy choices are influenced by drug availability and affordability. Loss to follow-up is yet another challenge; 22 of 54 identified patients could not be included due to incomplete records or non-compliance with follow-up, compromising both patient care and the strength of local data.

### Patterns of recurrence

Distant metastases dominated recurrence patterns in our cohort, consistent with published reports [19–26]. This reinforces the need for systemic therapy across all stages and suggests that local control alone is insufficient. The use of PCI in our series was clinician-driven and extrapolated from small cell lung cancer management [27]; current evidence does not support its routine use in NECC. Emerging molecular data, such as Kirsten Rat Sarcoma Viral Oncogene Homolog mutation–associated responses to mitogen activated protein kinase inhibitors, highlight the potential of targeted therapies and immunotherapy. However, molecular profiling remains largely inaccessible in LMICs.

### Strengths and limitations

#### Strengths

all cases were confirmed histologically by a dedicated gynaecologic oncopathologist using appropriate IHC panels. The study spans a 16-year period with a relatively long median follow-up of 42.6 months, which is notable for a rare cancer in a LMIC setting. Importantly, it includes real-world patients managed with diverse treatment strategies, thereby reflecting actual clinical practice in resource-constrained environments.

## Limitations

This study has several limitations. Its retrospective, single-centre with small sample size limits statistical power, affecting survival percentages. Loss to follow-up and incomplete data were observed in a substantial proportion of cases, reflecting real-world challenges in long-term outcome assessment. The lack of uniform imaging and absence of molecular profiling restrict the ability to derive detailed prognostic and therapeutic insights.

## Conclusion

NECC is a rare, aggressive cancer with poor survival across all stages. In our cohort, only a small subset of patients benefited from intensive multimodality treatment. Early diagnosis, timely initiation of systemic therapy and collaborative efforts are critical to improving outcomes, particularly in low-resource settings.

## Conflicts of interest

None declared.

## Funding

The authors have not declared a specific grant for this research from any funding agency in the public, commercial or not-for-profit sectors.

## Figures and Tables

**Table 1.** Baseline patient characteristics (n = 32).

Characteristic	Number/mean (total = 32)
Gravida <3 ≥3	11 (34.4%) 21 (65.6%)
Tobacco use	10
Presenting complaints	
Discharge PV	27 (90%)
Pain	16 (53.3%)
Post-menopausal bleeding PV	15 (50%)
Post coital bleeding	2 (6.7%)
DVT	1 (3.1%)

**Table 2.** Tumour and stage characteristics.

Characteristic	Number of patients (%)
Stage	
I	2 (6.2%)
II	2 (6.2%)
III	22 (68.7%)
IV	4 (12.5%)
Vaginal vault recurrence	2 (6.2%)
Tumor size	
≤4 cm	6 (18.8%)
>4 cm	26 (81.2%)
Pelvic node involvement	17 (53.1%)
Para-aortic node involvement	9 (29%)
Immunohistochemical staining	
AE1	20
Synaptophysin	32
Chromogranin	30
Neuron specific enolase	7
CD 56	2
Cytokeratin	9

**Table 3.** Outcomes of patients treated with primary surgery and adjuvant therapy (*n* = 5).

SI no	Age (years)	Stage	Type of surgery	Treatment (at our centre)	Tumour diameter	Site of recurrence	OS (month)	Status
1	50	IB3	RH+BSO+ BPLND+ PALND	Surgery – 2P + E-EBRT (50 Gy/25#) + PCI (24 Gy/12#) – 4P + E	7 × 5 × 4 cm	-	12	Dead
2	35	IB2	RH+BSO+ BPLND	Surgery - 2P+E – EBRT (50 Gy/25#) + PCI (24 Gy/12#) - 4P+E	3 × 4 cm	-	45	Dead
3	34	IIIC2r – recurrence	Outside RH+BSO+ BPLND (nodes, parametria, margins +) adjuvant not taken	Palliative whole brain RT + 1 cycle P+E	7.7 × 8 cm	Brain	6	Dead
4	32	I – recurrence	Outside – RH+BSO+ BPLND (adjuvant not taken)	6 P+E – Palliative EBRT (30 Gy/10#) –	5.7 × 5.2 cm	Pelvis	11	Dead
5	34	IIIC1p	Outside RH+BSO+ BPLND (pelvic node +)	EBRT 50 Gy/25# - 6P+E- 2#ICR	4.7 × 4.2 cm	Lost to follow up	-	Not known

BPLND = bilateral pelvic lymph node dissection; BSO = bilateral salpingo-ophorectomy; E = etoposide; EBRT = external beam radiotherapy; ICR = intracavitary radiotherapy (brachytherapy); P = cisplatin; PALND = para-aortic lymph node dissection; PCI = prophylactic cranial irradiarion; RH = radical hysterectomy

**Table 4.** Outcomes of patients treated with NACT followed by chemoradiation (*n* = 5).

S no	Age	Stage	Primary modality of treatment	Tumour diameter	OS (month)	Status
1	55	IIIC2	6 P+E -EBRT (50 Gy/25#), PCI (24 Gy/12#) + 2#ICR	7.1 × 6.5 cm	10	Dead
2	52	IIIC1r	1 P+E- EBRT (40 Gy/20#)-3#ICR - PCI (24 Gy/12#) - 3 P+E	6 × 7 × 8 cm	6	Dead
3	45	IIIC1 r	6 P+C + haemostatic RT to pelvis + PCI (24 Gy/12#)	2.2 × 3.3 cm	2	Dead
4	35	IVB	2 P+ E	5.5 × 5.7 cm	2	Dead
5	41	IVA	3 P+E	3.5 × 3.5 cm	Lost to follow up	Not known

**Table 5.** Stage-wise distribution of patients treated with definitive CCRT (*n* = 19).

Stage	*n* = 19
IIB	2 (10.5%)
IIIA	1 (5.2%)
IIIB	8 (42%)
IIIC1 r	4 (21%)
IIIC2 r	3 (15.7%)
IV A	1 (5.2%)

**Table 6.** Patients treated with palliative intent (*n* = 3).

S no	Age	Stage	Primary modality of treatment	OS (months)	Status
1	42	IIIB (femoral vein thrombus and brain metastasis)	EBRT (12 Gy/3#) + whole-brain RT	2	Dead
2	45	IVB (supraclavicular node+)	6 P+E - 1 gemcitabine + hemostatic RT (30 Gy/10#)	8	Dead
3	70	IIIC1 r (poor ECOG)	EBRT (30 Gy/10#)	6	Dead

**Table 7.** Univariate cox table (OS).

Variable	HR	95% CI	*p* value
Age ≥ 40	0.54	0.20–1.45	0.22
Stage III/IV	1.12	0.40–3.12	0.83
Parity ≥ 3	2.00	0.72–5.60	**0.19**
Pelvic LN+	0.86	0.35–2.12	0.74
Para-aortic LN+	0.84	0.32–2.23	0.73
Tumor size > 4 cm	0.89	0.34–2.32	0.81

Variables with *p* < 0.20 in univariate analysis were considered for inclusion in the multivariate Cox regression model. Statistical significance was defined as *p* < 0.05

**Table 8.** Multivariate cox table (OS).

Variable	HR	95% CI	*p* value
Age ≥ 40	0.53	0.20–1.42	0.21
Parity ≥ 3	2.03	0.73–5.67	0.18
